# Death of a Protein: The Role of E3 Ubiquitin Ligases in Circadian Rhythms of Mice and Flies

**DOI:** 10.3390/ijms231810569

**Published:** 2022-09-12

**Authors:** Osama Hasan Mustafa Hasan Abdalla, Brittany Mascarenhas, Hai-Ying Mary Cheng

**Affiliations:** 1 Department of Biology, University of Toronto Mississauga, Mississauga, ON L5L 1C6, Canada; 2 Department of Cell & Systems Biology, University of Toronto, Toronto, ON M5S 3G5, Canada

**Keywords:** circadian rhythms, E3 ubiquitin ligases, ubiquitin proteasome system, N-degron pathway, UBR4, protein degradation, clock proteins, *Drosophila*, suprachiasmatic nucleus

## Abstract

Circadian clocks evolved to enable organisms to anticipate and prepare for periodic environmental changes driven by the day–night cycle. This internal timekeeping mechanism is built on autoregulatory transcription–translation feedback loops that control the rhythmic expression of core clock genes and their protein products. The levels of clock proteins rise and ebb throughout a 24-h period through their rhythmic synthesis and destruction. In the ubiquitin–proteasome system, the process of polyubiquitination, or the covalent attachment of a ubiquitin chain, marks a protein for degradation by the 26S proteasome. The process is regulated by E3 ubiquitin ligases, which recognize specific substrates for ubiquitination. In this review, we summarize the roles that known E3 ubiquitin ligases play in the circadian clocks of two popular model organisms: mice and fruit flies. We also discuss emerging evidence that implicates the N-degron pathway, an alternative proteolytic system, in the regulation of circadian rhythms. We conclude the review with our perspectives on the potential for the proteolytic and non-proteolytic functions of E3 ubiquitin ligases within the circadian clock system.

## 1. Introduction

Life on Earth is strongly influenced by the 24-h day–night cycle, generated by the axial rotation of the planet. This cycle is accompanied by predictable environmental changes, such as daily variations in temperature, light, and food availability. Most organisms have evolved to anticipate such rhythmic occurrences in their physical environment by altering their behaviour and physiology in a similarly rhythmic fashion [1]. These biological rhythms, aptly termed circadian rhythms (from the Latin words “circa” (“approximately”) and “dies” (“a day”)), oscillate on a roughly 24-h cycle and arise from an organism’s endogenous timekeeping system, otherwise known as the circadian clock [2]. In mammals, the circadian system comprises a hierarchy of tissue-specific circadian clocks organized from the top-down and bottom-up [3,4]. The top-down arrangement occurs through the function of the master circadian pacemaker in the hypothalamus, the suprachiasmatic nucleus (SCN) [5]. As the central clock, the SCN encodes time-of-day information that it receives directly from the retina and conveys it to peripheral clocks to coordinate rhythms throughout the body [3,6]. In the bottom-up organization, peripheral clocks can feed information back to influence and regulate the activity of the SCN [3,4].

Circadian timekeeping is a cellular phenomenon that is based on transcription–translation feedback loops (TTFLs) of core clock genes and their protein products [2,7]. Through negative feedback mechanisms, TTFLs drive the rhythmic expression of core clock genes and circadian outputs with a near-24 h period. In addition to transcription, circadian rhythms are further regulated and fine-tuned by post-transcriptional, translational, post-translational, and degradative mechanisms [7]. The present review focuses strictly on a specific post-translational mechanism, ubiquitination, and its roles in circadian rhythms. For a detailed overview of the other modes of regulation, please refer to a recent review by Mendoza-Viveros et al. [7].

Ubiquitination (ubiquitylation) is the process by which a small, 76-amino acid protein, ubiquitin (Ub), is conjugated onto a substrate protein, most often at a lysine residue [8,9]. A substrate may be mono-ubiquitinated (i.e., a single Ub moiety on one residue), multi-ubiquitinated (i.e., multiple residues each carrying a ubiquitin), or poly-ubiquitinated (i.e., a single residue carrying a branched or straight chain of Ub proteins) [9,10]. The specific linkages of the ubiquitin or Ub chains, and the residue that is modified, determine the fate of the substrate protein, the most studied of which is degradation by 26S proteasomes [9,10]. These are multi-protein complexes present in the cytoplasm and nucleus that recognize ubiquitylated proteins and degrade them in a processive and ATP hydrolysis-driven fashion [11]. Ubiquitination may also affect the subcellular localization of a protein or its activity and function [12].

The conjugation of ubiquitin onto a substrate and the subsequent degradation of the ubiquitinated protein are mediated by the ubiquitin proteasome system (UPS). The UPS uses a series of three protein components, termed E1, E2, and E3, to covalently attach ubiquitin on the substrate [13]. In the first step, the Ub-activating enzyme, E1, catalyzes the linkage of Ub onto an internal cysteine residue of E1 by a thioester bond [13]. This is followed by the transfer of Ub from E1 to a cysteine residue of the Ub-conjugating enzyme, E2 [13]. In the third and final step, E2 interacts with a specific E3 protein that selectively binds substrates, enabling the transfer of Ub from E2 to a lysine residue of the substrate [13]. This process can repeat to conjugate additional Ub proteins onto the previous Ub at an internal lysine residue or the N-terminal methionine (M1), generating a polyubiquitin chain [9]. Ubiquitin has seven lysine residues (K6, K11, K27, K29, K33, K48, and K63) that are available as attachment sites for subsequent Ub proteins [9]. K48-linked chains are the most abundant linkages and direct the target protein to the 26S proteasome [9]. In humans, there are 2 genes that encode for E1, 38 for E2, and more than 600 for E3 [14]. The sheer number of E3 proteins (alternatively known as E3 ubiquitin ligases) reflects their important role in conferring substrate specificity to the UPS.

**Figure 1:**
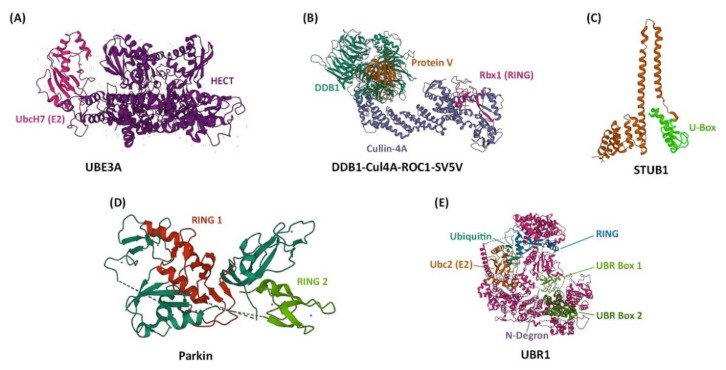
The structures of representative E3 ubiquitin ligases for each structural class. Atomic models were taken from the RCSB protein data bank (PDB, rcsb.org) and images were created using Mol* [20,21]. (**A**) UBE3A from humans, shown attached to the E2-ubiquitin-conjugating enzyme UbcH7. UBE3A is an example of a HECT type E3 ubiquitin ligase (PDB code 1C4Z) [22]. (**B**) DDB1–CUL4A-ROC1 from humans, shown attached to the non-structural V protein of the simian virus 5 (SV5-V). DDB1–CUL4A-ROC1 complex contains Ring-box-1 (Rbx1) and is an example of a RING finger type E3 ubiquitin ligase (PBD code 2HYE) [23]. (**C**) STUB1 from humans, representing a U-box type E3 ubiquitin ligase (PDB code 2C2L) [24]. (**D**) Parkin from brown rat, representing a RING-BetweenRING-RING type E3 ubiquitin ligase (PDB code 4K95) [25]. (**E**) UBR1 from yeast, shown in an initiating complex with ubiquitin, the E2-ubiquitin-conjugating enzyme Ubc2, and an N-Degron (indicated by a dashed line). All UBR proteins contain a UBR box, and UBR1 also contains a RING domain (PDB code 7MEX) [26].

There are four major structural classes of E3 Ub ligases: the HECT (Homologous to E6-AP Carboxyl Terminus) type E3s, the RING (Really Interesting New Gene) finger type E3s, the U-box type E3s, and the RBR (RING-BetweenRING-RING) type E3s (Figure 1) [15]. These E3 ligases interact physically with E2 through their HECT, RING finger, or U-box domains [15]. In the case of HECT E3 ligases, they contain a cysteine residue that can form a thioester bond with Ub originating from the E2 protein [15]. Neither the RING finger nor the U-box E3 ligases form an intermediate with Ub; instead, they facilitate the direct transfer of Ub from E2 to the substrate [15]. With the RBR E3 ligases, E2 binds to the RING1 domain of the ligase and Ub is then directly transferred from E2 to a cysteine residue in the RING2 domain [15]. The E3 ligases of the N-end rule pathway (recently renamed the N-degron pathway) represent a special family of E3s that specifically recognize substrate proteins by their destabilizing N-terminal residue [16]. Within this family is a subclass of E3s that are defined by the presence of a UBR box, a zinc finger-like domain [16]. E3 ligases of the N-degron pathway will be explored in further detail in Section 4. Most of the E3 ligases that have so far been implicated in circadian rhythms belong to the HECT and RING finger families. In addition to E3s, deubiquitinases (DUBs), which deconjugate Ub from a substrate protein, have also been shown to impact circadian rhythms by reversing the effects of ubiquitination [17,18,19].

In this review, we will focus on the effects of ubiquitination on the circadian systems of mammals, particularly mice (Section 2), and *Drosophila* (Section 3). Each section begins with a description of the relevant circadian system followed by a discussion of the roles of known E3 ligases. In Section 4, we discuss the emerging roles of E3 ligases of the N-degron pathway in circadian timekeeping. At the end of the review, we provide some perspectives regarding the roles of ubiquitination in circadian rhythms, beyond its well-characterized effects on protein stability.

## 2. Mammalian Circadian Rhythms and Ubiquitin Ligases

### 2.1. Overview of Circadian Rhythms in Mammals

#### 2.1.1. The Suprachiasmatic Nucleus

The SCN is a bilateral structure situated in the anterior hypothalamus, dorsal to the optic chiasm [27]. It consists of approximately 20,000 neurons, each functioning as autonomous circadian clocks [28]. The SCN is subdivided into ventrolateral and dorsomedial regions, otherwise referred to as the core and shell SCN, respectively [27]. The intrinsic period of individual SCN neurons can vary from ~22 h to 30 h [28,29]. However, intercellular coupling between SCN neurons results in their oscillating in synchrony with a significantly narrower period range [28,30]. Neurons in the ventrolateral SCN receive direct inputs from the retina; they also synthesize and secrete gastrin-releasing peptide (GRP) and vasoactive intestinal polypeptide (VIP) [27]. VIP is a neuropeptide vital to interneuronal coupling within the SCN, and by extension to the robustness of the central pacemaker [31]. Once released, both VIP and GRP signal to cells in the core and shell SCN [31,32]. Dorsomedial SCN neurons utilize a different neuropeptide, arginine vasopressin (AVP), to communicate with and couple to other clock neurons [31,32]. The inhibitory neurotransmitter, gamma-aminobutyric acid (GABA), is expressed by nearly all SCN neurons and has been shown to contribute to oscillator coupling and the refinement of circadian outputs [27,33].

As the intrinsic period of the SCN deviates slightly from 24 h, photic entrainment is required to maintain synchrony between the central pacemaker and environmental cycles. This involves the daily resetting of the clock by light, which synchronizes the SCN’s phase with the solar cycle [6,34]. In mammals, photic entrainment is fully dependent on the retina, and relies on the detection of changes in environmental light intensity [34,35]. As the master pacemaker, the SCN receives and integrates environmental photic signals from the retina, using them to synchronize its own neuronal clocks, after which the SCN can convey temporal information to peripheral clocks in the brain and body through synaptic and humoral mechanisms [35]. A direct projection from the retina to the SCN, known as the retinohypothalamic tract (RHT), transmits photic signals to the central clock via the actions of secreted glutamate and pituitary adenylate cyclase-activating peptide (PACAP) [36]. Within the retina, the non-image forming, intrinsically photoreceptive retinal ganglion cells (ipRGCs) are the main players in photic entrainment, using the photopigment melanopsin to detect light in the blue wavelength range, and transmitting the photic signal to the SCN via the RHT [37,38,39].

#### 2.1.2. The Mammalian Core Clock Machinery

**Figure 2:**
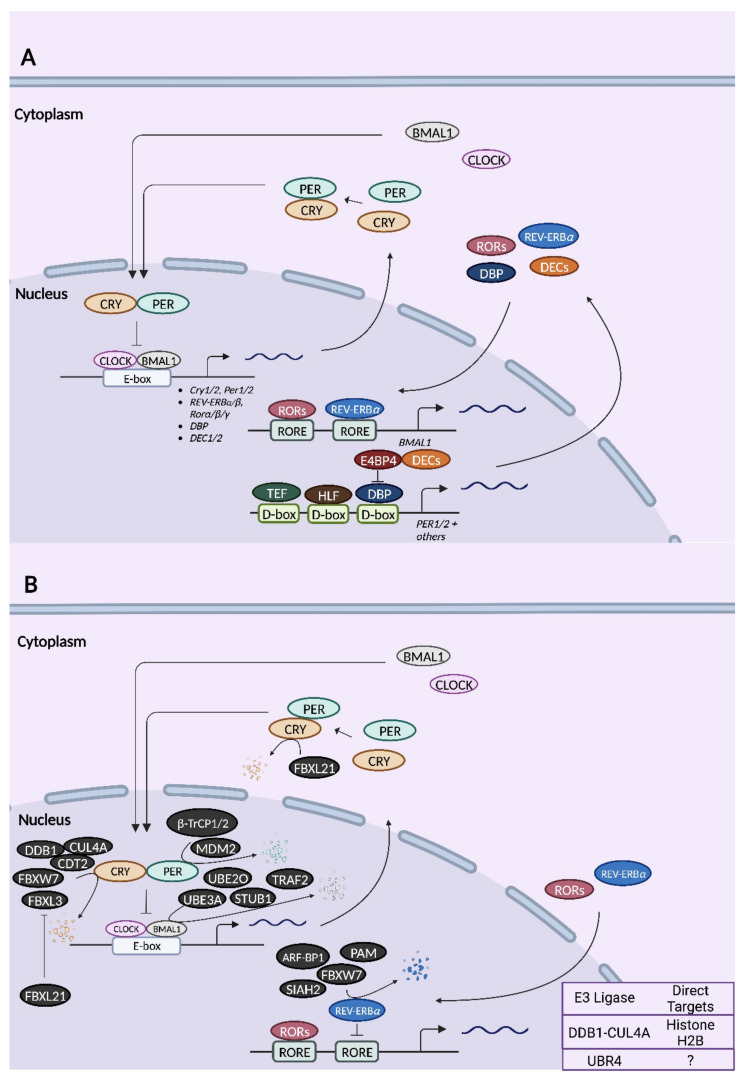
Schematic of mammalian circadian clock transcription–translation feedback loops (TTFLs) (**A**) and the relationships of known E3 ligases to core clock proteins (**B**). (**A**) In the primary TTFL of mammals, transcription factors CLOCK and BMAL1 heterodimerize and activate the transcription of target genes including *Cry1/2, Per1/2, Rev-Erbα/β, Rorα/β/γ*, *Dbp,* and *Dec1/2* by binding to the E-box elements in their promoters. Once translated, PER and CRY proteins accumulate in the cytoplasm before heterodimerizing and translocating to the nucleus, where they ultimately repress E-box-dependent transcription. In a secondary TTFL, RORα/*β/γ* binds to ROR elements (RORE) in the promoters of *Bmal1* and other genes to activate their transcription. In contrast, REV-ERB*α/β* binds to ROREs to repress transcription. Another auxiliary feedback loop involves DBP, TEF, and HLF, which bind to the D-box elements of *Per1/2* genes (among others), initiating D-box-mediated transactivation. Binding of E4BP4 at D-box elements results in transcriptional repression. (**B**) All mammalian E3 ubiquitin ligases that have been demonstrated to target a core clock protein for degradation are indicated in black. E3 ligases whose targets are either unknown or not core clock proteins are indicated in the chart in the bottom right corner. Except for FBXL3 and FBXL21, the site of action (nucleus vs. cytoplasm) for all other E3 ligases is unknown. For illustrative purposes, these E3 ligases are shown as acting in the nucleus.

Circadian TTFLs operate on the principle of negative feedback inhibition, whereby elements in the positive limb of the feedback loop drive the expression of elements within the negative limb, eventually shutting off their own expression until the cycle begins anew ~24 h later [40]. Within the primary TTFL of mammals, the core clock genes, *Clock* and *Bmal1* (officially known as *Arntl*), represent the positive elements, whereas *Period1 (Per1)*, *Period2 (Per2)*, *Cryptochrome1 (Cry1),* and *Cryptochrome2 (Cry2)* represent the negative elements (Figure 2) [41,42,43,44,45]. The positive limb is most active during the subjective day, when the helix-loop-helix transcription factors, CLOCK and BMAL1, heterodimerize and bind to the E-box *cis*-regulatory elements of the *Per* and *Cry* gene promoters, activating their transcription [2,7]. Following their translation and accumulation in the cytoplasm, PER and CRY proteins heterodimerize and translocate to the nucleus [2,7]. This initiates the negative limb, as PER and CRY repress their own transcription, either by binding to CLOCK/BMAL1 and blocking E-box-mediated transactivation or by displacing CLOCK/BMAL1 from E-boxes [46]. The two modes of repression are differentially mediated by PER and CRY [46,47,48]. In the “blocking”-style repression, CRY1 binds to CLOCK-BMAL1-E-box complexes independently of PER to inhibit transactivation: the repression occurs even as CLOCK and BMAL1 remain bound to the E-box [46,47,48]. In the “displacement”-style repression, PER2, in the presence of CRY, displaces CLOCK/BMAL1 from the E-boxes [46,47,48]. PER proteins on their own have no repressive activity towards CLOCK/BMAL1 [46]. Eventually, PER and CRY are degraded by the proteasomal pathway, allowing CLOCK/BMAL1 to once again occupy E-box elements and activate gene expression [2,7].

Alongside the primary TTFL there exist ancillary feedback loops that stabilize and fine-tune the clock, connecting it to other cellular pathways [2,45]. An important secondary TTFL found in mammals is the one involving REV-ERB nuclear receptors and retinoic acid receptor-related orphan receptors (RORs), both acting on ROR elements (ROREs) in gene promoters [2,45,49,50]. The *Bmal1* promoter contains two ROREs, which lead to transcriptional activation when bound by RORα, RORβ, and RORγ, and transcriptional repression upon the binding of Rev-Erbα and Rev-Erbβ [2,49,50,51]. BMAL1 controls the expression of its own activators and repressors through E-box-mediated transactivation of *Rorα/β/γ* and *Rev-Erbα/β*, driving their rhythmic expression [2]. Yet another ancillary TTFL involves the three PAR domain-basic leucine zipper (PAR bZIP) transcription factors, albumin D site-binding protein (DBP), thyrotroph embryonic factor (TEF), and hepatic leukemia factor (HLF), and the bZIP transcription factor, nuclear factor interleukin-3 regulated (NFIL3/E4BP4), acting at D-box elements [52]. D-box sequences are present in potentially thousands of genes including *Per1* and *Per2* [52,53,54]. The binding of DBP triggers D-box-mediated transactivation, while NFIL3/E4BP4 binding, in association with another circadian transcription factor, basic helix-loop-helix family member e41 (BHLHE41/DEC2), results in repression [55]. The *Dbp*, *Nfil3*/*E4bp4,* and *Dec1/2* genes are themselves under E-box regulation [56,57].

### 2.2. Mammalian Ubiquitin Ligases

#### 2.2.1. β-TrCP1 (FBXW1) and β-TrCP2 (FBXW11)

Beta-transducin repeat-containing proteins (β-TrCP) were the first E3 ubiquitin ligases to be implicated in the regulation of the clock machinery. β-TrCP is an F-box protein of the Fbws class, characterized by the presence of an F-box motif and tandem WD40 repeats [58]. F-box proteins are the substrate recognition subunits of the SCF (Skp1-Cullin 1-F-box protein) family of E3 ubiquitin ligases [58]. Casein kinase 1 (CK1)-mediated phosphorylation of PER2 was shown to trigger an association between β-TrCP and PER2 [59]. The interaction between β-TrCP and PER2 is also facilitated by the SUMOylation of PER2 by SUMO2 [60]. The expression of mutant β-TrCP that lacks an F-box inhibits the degradation of phosphorylated PER2 [59]. In another study, β-TrCP1 and β-TrCP2 were identified as binding partners of PER1 [61]. These interactions are dependent on the phosphorylation of PER1 by CK1ε [61]. Furthermore, the silencing of β-TrCP1 stabilizes PER1 and inhibits CK1ε-induced PER1 degradation [61]. Cell-free assays showed that SCF complexes containing β-TrCP are capable of ubiquitinating PER1 [61]. In NIH-3T3 fibroblasts, the knockdown of *β-TrCP1* or the expression of dominant-negative β-TrCP1 elicits a lengthening of molecular oscillations [62]. PER2 mutants that are unable to interact physically with β-TrCP1/2 exhibit severely disrupted or damped rhythms in fibroblasts [62]. However, β-TrCP1 appears to be dispensable for circadian rhythms at the behavioural level, as β-TrCP1 knockout mice are phenotypically normal with respect to period length and light-induced phase shifts [63]. On the other hand, introducing the PER2 S478A mutation, which can no longer be phosphorylated by CK1δ/ε and thus cannot recruit β-TrCP1/2, results in a dramatic lengthening of the behavioural period in knock-in mice and the accumulation of PER2 protein in the nucleus and cytoplasm of the liver, suggesting that β-TrCP2 may compensate for the loss of β-TrCP1 [64]. In line with this, inducible β-TrCP2 knockout mice exhibit a dramatic circadian phenotype characterized by unstable behavioural rhythms and period variability under constant darkness (DD) [65]. The fact that the ubiquitination of PER2 is still observed in the absence of β-TrCP1 and β-TrCP2 indicates that PER2 is a substrate of other E3 ligases [65]. β-TrCP1/2 may also influence the circadian clock by ubiquitinating other clock proteins such as DEC1 and targeting them for proteasomal degradation [66].

#### 2.2.2. Mouse Double Minute 2 Homolog (MDM2)

MDM2 is a RING finger type E3 ligase that serves as a scaffold, bringing E2 enzymes to protein substrates for ubiquitination [67]. PER2 can form trimeric complexes with MDM2 and its substrate, p53 [68]. However, PER2 and MDM2 can directly associate with each other independently of p53 [68]. Furthermore, MDM2 binds to PER2 at the latter’s PAS domain and an inner region that undergoes extensive phosphorylation [68]. CK1δ/ε-mediated phosphorylation of PER2 is not necessary for either MDM2 binding or MDM2-dependent ubiquitination of PER2 [68]. MDM2 destabilizes PER2 and appears to work cooperatively with β-TrCP to control the abundance of PER2 during the rising and falling phases of the protein’s circadian cycle [68]. The knockdown of *mdm2* extends the circadian period in murine embryonic fibroblasts (MEFs), whereas the overexpression of *mdm2* shortens it [68].

#### 2.2.3. FBXL3

FBXL3 was first reported in three sister studies in 2007 as an E3 ubiquitin ligase for CRY proteins [69,70,71]. By chemical-induced mutagenesis, two mutations in FBXL3 were identified that dramatically lengthen the circadian period: the C358S substitution termed *afterhours (Afh)* and the I364T mutation termed *overtime (Ovtm)* [70,71]. In both *Afh* and *Ovtm* mutant mice, CRY1/2 protein levels are not appreciably altered but PER1/2 abundance is strongly suppressed, suggesting that the stabilization of CRY1/2 in the presence of lower E3 ligase activity is compensated for by a reduction in E-box-dependent transcription of *Cry1/2* genes [70,71]. FBXL3 binds specifically to CRY1 and CRY2 but not to other core clock proteins and promotes their degradation by proteasomes [69,71]. FBXL3 was also shown to promote CRY2 ubiquitination in an F-box-dependent manner, suggesting a direct effect of FBXL3 on CRY2 [69]. Both the *Afh* and *Ovtm* mutations reduce the rate of CRY1/2 degradation; in the case of the *Afh* mutation, this was attributed to the reduced binding of FBXL3(*Afh*) to CRY proteins and reduced catalytic activity [69,71]. As a consequence of their effects on CRY stability, these mutations dampen the amplitude of circadian gene expression [70,71].

Crystal structure analysis revealed a bipartite interaction between FBXL3 and CRY2, in which the C-terminal tail of FBXL3 occupies the FAD-binding pocket of CRY2, and the leucine-rich repeat (LRR) domain of FBXL3 is a secondary site of contact for CRY2 at three key structural motifs [72]. As these interactions occur in the absence of FAD or PER binding to CRY2, either of these factors can disrupt the FBXL3–CRY2 complex or prevent its formation [72]. The formation of FBXL3–CRY complexes is required for the recruitment of SKP1 and CUL1, thereby forming the fully functional SCF complex [73]. This substrate-dependent formation of SCF complexes appears to be specific for FBXL3 and is not observed with FBXL21 [73].

CRY2 binding may also recruit FBXL3 to other substrates such as c-MYC and TLK2 to induce their ubiquitination and subsequent degradation, thereby linking the clock to proteolysis in other physiological systems [74]. In addition to CRY1/2, FBXL3 has also been shown to physically associate with REV-ERBα in mouse livers [75]. REV-ERBα recruits FBXL3 to RORE sites, where it derepresses gene expression by inhibiting the actions of REV-ERBα:HDAC3 complexes [75].

#### 2.2.4. FBXL21

The F-box protein FBXL21 is the closest homologue of FBXL3 [58]. Initial studies revealed that FBXL21 is expressed in the brain and neuroendocrine tissues of sheep, and physically associates with ovine CRY1 [76]. *Fbxl21* harbours functional E- and D-box elements within its promoter, resulting in high and rhythmic expression of the gene in the ovine and murine SCN [76]. Furthermore, FBXL21 overexpression abrogates the repressive effects of CRY1 on CLOCK/BMAL1-mediated transcription [76]. A subsequent study showed that the *past-time* (*Psttm*) mutation, which is a missense mutation (G149E) in the *Fbxl21* gene, significantly shortens the circadian period and antagonizes the period-lengthening effects of FBXL3(*Ovtm*) [77]. FBXL21 binds to CRY1/2 with a higher affinity than FBXL3, effectively outcompeting FBXL3 [77]. In the nucleus, where both FBXL21 and FBXL3 are present, FBXL21 sequesters CRY proteins from FBXL3 and protects them from FBXL3-induced proteasomal degradation [77]. In the cytosol, where FBXL3 is absent, FBXL21 triggers the slow degradation of CRY1/2 [77]. It was further shown that FBXL21 preferentially forms SCF complexes in the cytoplasm but not in the nucleus [77]. These collective observations are consistent with the different potencies of the two FBXL homologs in CRY destabilization, where FBXL21 appears to be a weaker E3 ligase for CRY than FBXL3 [77]. The effects of FBXL21 on the stability of nuclear and cytoplasmic CRY are such that *Psttm* mutant mice have altered core clock gene expression that is characterized by a higher expression of E-box-regulated genes [77]. Although a different group confirmed many of these findings, including the antagonism between FBXL3 and FBXL21, the stabilization of CRY by FBXL21, and the preferential localization of FBXL21 in the cytoplasm, in their case, *Fbxl21*-deficient mice did not show a circadian period phenotype, unlike the *Psttm* mutants [78].

#### 2.2.5. DDB1–CUL4A and DDB1–CUL4A–CDT2

The DDB1–CUL4A–CDT2 E3 ubiquitin ligase complex has been shown to target CRY1 for degradation [79,80]. In vitro assays revealed that DDB1–CUL4A–CDT2 directly ubiquitinates CRY1 at Lys-585, marking the protein for proteasomal degradation [80]. CRY1 physically binds to CDT2 and the silencing of *Cdt2* prevents complex formation between CRY1 and DDB1–CUL4A [80]. In mouse hepatoma cells, the knockdown of *Ddb1* or overexpression of the ubiquitination-defective mutant CRY1 K585A enhances CRY1 stability and increases the amplitude of circadian oscillations as measured by a *Bmal1-Luc* reporter [80]. In a subsequent study, liver-specific *Ddb1* knockout mice were shown to have impairments in hepatic gluconeogenesis but were protected from high-fat-diet-induced hyperglycemia [79]. These effects are due to elevated levels of CRY1, which binds to the FOXO1 transcription factor and promotes its ubiquitination and degradation [79]. In turn, the lower abundance of FOXO1 in *Ddb1* knockouts suppresses gluconeogenic gene expression [79]. Besides CRY1, DDB1–CUL4A has been shown to interact with CLOCK-BMAL1 [81]. These circadian transcription factors bind to an adaptor protein of the DDB1–CUL4A complex, WD repeat-containing protein 76 (WDR76) [81]. Through this interaction, CLOCK-BMAL1 recruits DDB1–CUL4A to E-boxes of the *Per1* and *Per2* genes as well as other circadian genes [81]. DDB1–CUL4A enhances the monoubiquitination of histone H2B at E-box sites, which subsequently inhibits CLOCK-BMAL1 binding while promoting the association with PER complexes [81].

#### 2.2.6. FBXW7

FBXW7 is an F-box protein of the Fbws class [58]. Several studies have implicated the involvement of FBXW7 in the regulation of circadian rhythms by targeting different proteins. In mice injected with renal carcinoma cells, the expression of FBXW7 exhibits circadian oscillations in the tumours, driven by DBP, which binds to and transactivates *Fbxw7* in a rhythmic fashion [82]. The mammalian target of rapamycin (mTOR), a key protein in cell growth and the circadian control of translation, oscillates in anti-phase to FBXW7 protein [82]. A prior study demonstrated that FBXW7 ubiquitinates mTOR and targets it for proteasomal degradation [83]. A separate study revealed that FBXW7 binds to CRY2 in colorectal cancer cells, potentially through a direct interaction between the degron motif of CRY2 and the narrow face of the WD40 domain of FBXW7 [84]. It was further shown that FBXW7 destabilizes CRY2 by promoting its ubiquitination and subsequent proteasomal degradation [84]. REV-ERBα is another identified target of FBXW7 [85]. FBXW7 was demonstrated to physically interact with REV-ERBα, enhancing its ubiquitination and destabilizing it [85]. Cyclin-dependent kinase 1 (CDK1)-mediated phosphorylation of REV-ERBα at Thr-275 is required for its recognition by FBXW7 [85]. The amplitude of circadian gene expression is suppressed when *Fbxw7* is silenced in cultured cells or ablated in mouse livers [85]. The deletion of *Fbxw7* specifically in the liver alters the hepatic circadian transcriptome and disrupts whole-body lipid and glucose metabolism [85].

#### 2.2.7. TNF Receptor-Associated Factor 2 (TRAF2)

The ubiquitin ligase TRAF2 was initially identified as a CRY1-binding protein in a high-throughput yeast two-hybrid screen [86]. However, a subsequent study showed that TRAF2 overexpression does not alter CRY1 abundance, suggesting that the interaction between TRAF2 and CRY1 does not lead to the degradation of the latter [87]. The same study also revealed that TRAF2 physically binds to BMAL1 and reduces its abundance [87]. The physical association is mediated by the zinc finger domain of TRAF2 and not by the TRAF domain, the canonical substrate recognition site [87]. Furthermore, the deletion of the RING domain of TRAF2 stabilizes BMAL1 protein, indicating that the effects of TRAF2 are dependent on its ubiquitin ligase activity [87]. Consistent with these results, the overexpression of TRAF2 promotes the ubiquitination of BMAL1 and its degradation by proteasomes [87]. The TRAF2-dependent reduction in BMAL1 abundance ultimately attenuates E-box-mediated transcription and dampens *Per1* oscillations [87].

#### 2.2.8. STIP1 Homology and U-Box-Containing Protein 1 (STUB1)

STUB1 was identified in a mass spectrometric analysis of BMAL1-binding partners [88]. This interaction is selective for BMAL1 and is not observed with CLOCK [88]. The overexpression of wild-type STUB1, but not of a catalytically inactive form, reduces the abundance of BMAL1 protein in HEK293T cells, indicating that STUB1 affects BMAL1 stability through its ubiquitin ligase activity [88]. Along these lines, STUB1 catalyzes the K48-linked polyubiquitination of BMAL1, which is associated with proteasomal degradation [88]. STUB1 is primarily localized to the cytosol, but upon oxidative stress, it translocates to the nucleus where it can destabilize BMAL1 to attenuate cellular senescence [88].

#### 2.2.9. UBE3A

UBE3A is a HECT-domain-containing ubiquitin ligase expressed in multiple tissues, including the SCN [89]. It is also the causative gene for the neurodevelopmental disorder Angelman Syndrome (AS), in which UBE3A expression is absent due to the loss of the maternal allele amid the normally silenced paternal allele [90]. There is evidence for and against paternal imprinting of the *Ube3a* gene in SCN neurons [91,92]. Sleep disturbances are one of the symptoms of AS, which include delayed development, intellectual disability, impaired speech, and motor dysfunction [93]. Ablating the maternal copy of *Ube3a* in mice consistently disrupts sleep homeostasis [89,92,94]. However, one study found that this mutation also lengthens the circadian period under constant darkness (DD), accelerates recovery from jetlag (i.e., mice re-entrain more rapidly to an advanced light–dark schedule), and suppresses locomotor activity under constant light (LL), whereas another study found no effect on circadian period [89,95]. At the molecular level, the activation of UBE3A in mouse embryonic fibroblasts (MEFs) by the viral oncogenes E6/E7 triggers the ubiquitination of BMAL1 and a reduction in its protein abundance through proteasomal degradation, ultimately leading to a loss of circadian rhythms [96]. These effects are direct, as UBE3A physically binds to, polyubiquitinates, and destabilizes BMAL1 [95,96]. Consistent with these observations, the loss of the maternal allele of *Ube3a* elevates BMAL1 abundance in the murine hypothalamus [95]. Lastly, even in the absence of E6/E7-mediated transformation, the endogenous activity of UBE3A is required for maintaining robust rhythms of *Per2* expression in MEFs [96].

#### 2.2.10. UBE2O

UBE2O is a ubiquitin-conjugating enzyme with hybrid E2/E3 activity [97]. Initially identified from a mass spectrometry screen for BMAL1-binding proteins, UBE2O was shown to physically associate with BMAL1 in mouse Neuro2a cells and whole brain tissue [98]. The overexpression of UBE2O reduces the levels of endogenous BMAL1 in HEK293T cells in a dose-dependent fashion, whereas its knockdown elevates BMAL1 abundance [98]. These effects are specific to BMAL1 and are not observed with CLOCK [98]. UBE2O was further shown to ubiquitinate BMAL1 and dramatically reduce its half-life [98]. The conserved region 2 (CR2) domain of UBE2O is essential for BMAL1 binding and ubiquitination [98]. The effects of UBE2O on BMAL1 stability lead to attenuated BMAL1 transcriptional activity when UBE2O is overexpressed and a higher amplitude of *Per2* rhythms when it is silenced [98].

#### 2.2.11. Seven in Absentia 2 (SIAH2)

SIAH2 was identified in a functional screen for REV-ERBα-directed ubiquitin ligases [99]. The overexpression of SIAH2 selectively destabilizes REV-ERBα and REV-ERBβ, but not the other proteins tested, including PER1, PER2, CRY1, and CLOCK [99]. In contrast, the SIAH2 paralog, SIAH1, does not influence REV-ERB stability [99]. More importantly, SIAH2 was shown to physically interact with REV-ERBα/β and promote its ubiquitination [99]. Ablating the RING domain of SIAH2 interferes with its ability to destabilize REV-ERB, indicating the importance of its catalytic function as an E3 ubiquitin ligase [99]. In synchronized U2OS cells, the knockdown of *Siah2* slows the turnover of REV-ERBα, thereby affecting its rhythms and the expression of its target genes, as well as lengthening the circadian period [99]. However, in mouse models, the absence of *Siah2* does not impact REV-ERBα protein rhythms, likely as a result of compensation by other E3 ligases, although other clock genes such as *Bmal1* and *Per2* are moderately affected at the transcript level [100]. In a surprising twist, *Siah2* deficiency disrupts the circadian hepatic transcriptome only in female mice [100]. Global circadian gene expression in the female liver is phase-advanced by ~9 h such that genes that are normally expressed during the night now peak in the daytime [100]. The underlying cause for this sexual dimorphism remains unknown.

#### 2.2.12. ARF-BP1 and PAM (Myc-BP2)

ARF-BP1 (also known as HUWE1) and PAM are HECT and RING finger type E3 ligases, respectively [101,102]. Both were co-purified with REV-ERBα in the presence of lithium chloride, an inhibitor of glycogen synthase kinase 3 beta (GSK3β) and a known inducer of REV-ERBα degradation, and subsequently identified by mass spectrometry [103]. Co-overexpression of ARF-BP1 and PAM strongly suppresses REV-ERBα protein levels, whereas their simultaneous depletion stabilizes REV-ERBα [103]. Furthermore, ARF-BP1 and PAM exclusively promote the K48-linked polyubiquitination of REV-ERBα, targeting the protein for degradation [103]. The knockdown of both ARF-BP1 and PAM in hepatoma cells results in a higher amplitude of REV-ERBα protein rhythms and consequently a lower expression and oscillatory amplitude of *Bmal1* [103].

## 3. Fly Circadian Rhythms and Ubiquitin Ligases

### 3.1. Overview of Circadian Rhythms in Flies

#### 3.1.1. The *Drosophila* Clock Network

In *Drosophila*, the central pacemaker function is mediated by a coupled network of ~150 clock neurons [104]. These neurons form seven anatomical clusters: the four large and the four small ventrolateral neurons (l-LN_v_s and s-LN_v_s, respectively), the lateral posterior neurons (LPNs), the dorsolateral neurons (LN_d_S), and three classes of dorsal neurons (DN1, DN2, and DN3) [104]. Each cluster expresses a distinct combination of molecular markers and neuropeptides and influences different facets of clock-controlled behaviour [105]. The l-LN_v_s and s-LN_v_s are the only neurons within the network to express the neuropeptide, pigment-dispersing factor (PDF), which is homologous to mammalian VIP [106]. There is a lone, fifth s-LN_v_ that is devoid of PDF and is frequently considered with the LN_d_s [104]. The s-LN_v_s are recognized as the master pacemaker neurons, as they are essential for the generation of behavioural rhythms under DD [107,108,109]. In addition to driving DD rhythms, the s-LN_v_s in *Drosophila* are responsible for the early morning activity displayed by this crepuscular organism, earning them the moniker of morning cells (M-cells) [110,111]. Time-of-day information is conveyed by s-LN_v_s to the rest of the network through the rhythmic release of PDF from their dorsal projections [106,112]. In flies, there are three separate pathways that mediate circadian photoreception [113]. The most studied mechanism involves CRYPTOCHROME, which functions as a blue light-sensitive photoreceptor in *Drosophila* [114]. The translucent nature of the fly’s body and the fact that CRY is present in all clock cells mean that CRY-mediated photoentrainment can occur in a cell-autonomous manner in both central and peripheral clocks [115]. The Hofbauer–Buchner eyelets, as well as the compound eye and ocelli, constitute the remaining pathways for circadian photoreception [116,117,118].

#### 3.1.2. Core Clock Machinery in Drosophila

**Figure 3:**
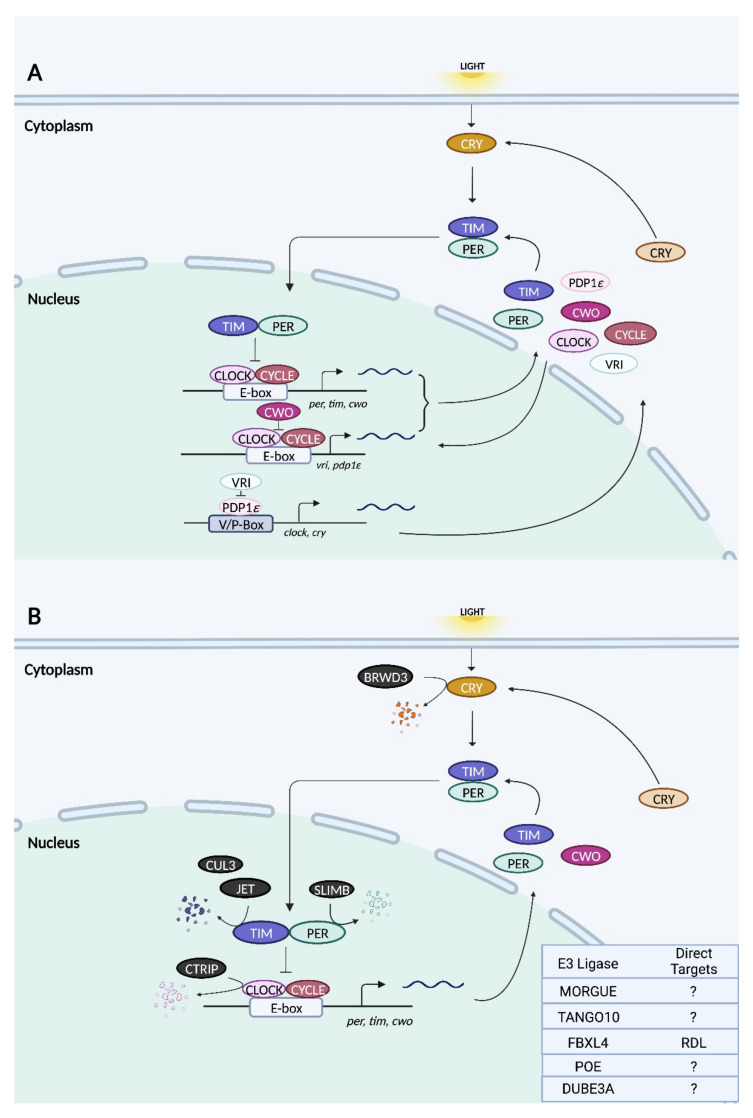
Schematic of circadian transcription–translation feedback loops (TTFLs) (**A**) and the relationships of known E3 ligases to core clock proteins in *Drosophila* (**B**). (**A**) In the primary TTFL of flies, transcription factors CLOCK and CYCLE heterodimerize and trigger E-box-mediated transactivation of target genes including *per, tim, vri, pdp1ε,* and *cwo.* Once translated, PER and TIM heterodimerize and accumulate in the cytoplasm. Following their translocation to the nucleus, PER:TIM complexes repress their own E-box-mediated transcription through association with CLOCK:CYCLE. In a secondary TTFL, the proteins VRI and PDP1ε exhibit opposing functions, where the former inhibits *clock* transcription at the V/P box region, while the latter activates it. Lastly, CWO competes with CLOCK:CYCLE for E-box binding, thereby repressing transcription in yet another feedback loop. *Drosophila* CRY is a photoreceptor that aids in TIM degradation upon light activation. (**B**) All *Drosophila* E3 ubiquitin ligases that have been demonstrated to target a core clock protein for degradation are indicated in black. E3 ligases whose targets are either unknown or not core clock proteins are indicated in the chart in the bottom right corner. The site of action (nucleus vs. cytoplasm) for all E3 ligases is not known. In the figure, the localization of each E3 ligase is for illustrative purposes only.

The architecture of the molecular clock machinery in *Drosophila* is broadly similar to that in mammals, with some notable exceptions. In the primary feedback loop, CYCLE (CYC), the homolog of mammalian BMAL1, partners with CLOCK (CLK) to activate the transcription of genes containing E-box elements (Figure 3) [119,120,121]. Two such genes are *period (per)* and *timeless (tim)*, whose protein products heterodimerize and accumulate in the cytoplasm [122,123,124]. Following a timed delay, PER:TIM complexes translocate to the nucleus, where they associate with CLK:CYC to repress E-box-dependent transcription, thereby closing the feedback loop [125,126]. The regulated degradation of PER and TIM leads to the derepression of CLK:CYC and the initiation of the next round of E-box-mediated transcription [120]. Unlike mammalian CRY, which couples with PER to form the negative elements of the TTFL, *Drosophila* CRY functions as a blue light-responsive photoreceptor whose primary role is in light-induced clock resetting [114]. Upon light activation, CRY interacts physically with TIM and promotes its degradation [127,128].

Additional feedback loops exist to fine-tune the pacemaking of the primary TTFL. One such loop regulates the rhythmic transcription of *clock* with a pair of opposing bZIP transcription factors: PDP1ε activates *clk* transcription, whereas VRILLE inhibits it [129,130]. Even though *vrille* and *pdp1ε* are both E-box-regulated genes, their protein products peak at different phases, with VRILLE peaking prior to PDP1ε; this allows their opposing actions on *clk* to be temporally segregated [129]. In a third feedback loop, the product of the E-box-dependent gene *clockwork orange (cwo)* acts antagonistically to CLK:CYC by competing for E-box binding and repressing transcription [131,132].

As is the case in mammals, the molecular clock machinery in *Drosophila* is regulated by the ubiquitin–proteasome pathway [120]. Regulated proteolysis has been noted for CLK, PER, TIM, and CRY [120]. A recent study also hinted at potential, non-proteolytic functions of ubiquitination [133]. In the next section, we explore the functions of the ubiquitin ligases that have been implicated thus far in circadian rhythms of fruit flies.

### 3.2. Fly Ubiquitin Ligases

#### 3.2.1. SLIMB

SLIMB is the *Drosophila* homolog of β-TrCP and the first E3 ubiquitin ligase to be implicated in the regulation of the circadian clock machinery in flies. *Slimb* mutants display complete arrhythmicity under DD whereas the restoration of *slimb* expression specifically in the LN_v_s rescues the phenotype [134]. In contrast, the LN_v_-targeted overexpression of *slimb* on a wild-type background results in period lengthening [134]. Another group found that the ubiquitous overexpression of *slimb* using the *tubulin* promoter renders the flies arrhythmic under DD, as does the overexpression of a *slimb* mutant lacking the F-box in *tim*-expressing clock cells [135]. *Slimb*-deficient flies are characterized by low-amplitude PER and TIM protein rhythms in head extracts as well as the persistence of the hyperphosphorylated forms of both proteins throughout the entire circadian cycle, suggesting a defect in their rhythmic turnover [134]. SLIMB preferentially associates with PER following the phosphorylation of the latter protein by DOUBLETIME (DBT), the fly homolog of CK1ε, and triggers PER degradation [135]. The silencing of *slimb* in S2 cells promotes the accumulation of hyperphosphorylated PER but only in the presence of DBT [135]. Thus, DBT-mediated phosphorylation of PER likely creates a signal that leads to the recruitment and binding of SLIMB. It has also been suggested that clock- and light-dependent degradation of PER and TIM may occur by different mechanisms, as *slimb* deficiency does not affect the rhythmic cycling of either protein in fly head extracts [134].

With respect to the physical association between SLIMB and PER, the first 100 amino acids of PER are required for SLIMB binding, as is the phosphorylation of PER at Ser-47 by DBT [136]. Phospho-Ser47 collaborates with other phosphorylation events of nearby residues to create a high-affinity SLIMB binding site, which is distinct from the major phospho-cluster spanning amino acids 583–596 of PER [136]. Both the phosphorylation of PER at Ser-47 and PER–SLIMB association occur near the end of the subjective night, indicating that SLIMB-directed degradation controls the downswing of nuclear PER levels at the end of the circadian cycle [136]. Mutations in *per* that abrogate (S47A) or mimic (S47D) Ser-47 phosphorylation lengthen or shorten, respectively, the behavioural period, suggesting that DBT-triggered degradation of PER by SLIMB is a key determinant of circadian clock speed [136].

#### 3.2.2. DUBE3A

The involvement of DUBE3A in circadian rhythms was first demonstrated in *Drosophila* [137]. A null mutation of the homologous gene *dube3a* results in weakly rhythmic or arrhythmic flies [137]. These phenotypes are apparent in older female flies (18–21 days) and in both young (4–7 days) and old male flies [137]. Arrhythmic behaviour under DD is recapitulated by the RNAi-mediated silencing of *dube3a* specifically in PDF neurons [96]. Interestingly, the overexpression of DUBE3A in these cells also results in arrhythmicity, suggesting that maintaining appropriate levels of DUBE3A is essential for the regulation of circadian rhythms [96]. DUBE3A is broadly expressed in the central nervous system of flies and is mainly localized to the cytoplasm [137]. The mechanistic underpinnings of DUBE3A’s effects on circadian rhythms remain to be elucidated.

#### 3.2.3. CTRIP

CTRIP (short for *circadian trip*) is a HECT type E3 ligase in fruit flies with homology to mammalian *trip12* [138]. In the fly brain, *ctrip* expression is strong and highly enriched in PDF neurons, but there is no evidence that it oscillates in a circadian fashion [139]. The deletion of the first five exons of *ctrip* disrupts the larval clock such that levels of PER, TIM, and CLK are elevated in the s-LN_v_s and the period of their oscillations is lengthened [139]. The silencing of *ctrip* in *tim*-expressing clock cells produces similar effects in the s-LN_v_s of adult flies, slowing the pace of behavioural rhythms [139]. Cell culture experiments revealed that a knockdown of *ctrip* attenuates CLK degradation, which is consistent with the notion that CLK is a substrate of CTRIP [139]. Furthermore, the phosphorylated forms of PER and TIM persist through the subjective day when *ctrip* is downregulated, suggesting that their degradation may also be impaired [139]. In a *per*-null background, *ctrip* silencing continues to enhance CLK levels whereas its effects on TIM are abolished [139]. It is therefore possible that CLK and PER, but not TIM, are both independent substrates of CTRIP.

#### 3.2.4. JET

JET was identified in a study that characterized fly mutants with strong rhythmic behaviours under LL [128]. A putative component of SCF complexes, JET has an N-terminal F-box and seven LRR domains important for protein–protein interactions [128]. Two mutations found within adjacent LRRs buffer the circadian clock against the disruptive effects of constant light, allowing for the persistence of behavioural rhythms [128]. In addition, *jet* mutants are less responsive to the phase-shifting effects of a nocturnal light pulse and require more days to re-entrain to a shifted light–dark schedule, effects that are rescued by over-expressing wild-type *jet* in clock cells [128]. These phenotypes are due to an impairment in light-induced TIM degradation in *jet* mutants [128]. JET-dependent TIM degradation requires the presence of CRY and is mediated by the proteasome [128]. Wild-type JET physically binds to TIM and ubiquitinates it, whereas mutant JET is less efficient catalytically [128].

In addition to TIM, JET was later shown to induce CRY degradation [140]. Yeast 2-hybrid experiments revealed light-dependent, direct binding between JET and CRY [140]. In the presence of light, mutations or deficiency in *jet* results in a greater abundance of CRY in fly heads, a finding that is recapitulated in S2 cells [140]. TIM appears to be preferred over CRY as a substrate of JET, which is possibly rate-limiting; thus, in the presence of TIM, CRY is protected from light- and JET-dependent degradation [140]. The study suggests that JET mediates the degradation of both TIM and CRY in a sequential manner due to differences in their binding affinities [140]. Light-induced conformational changes in CRY increase its affinity for JET [141].

In an interesting twist, *jet* mutations also rescue the arrhythmic phenotypes of flies that are mutant for *slowpoke (slo)* and *dyschromic (dysc)*, which encode a voltage-gated potassium channel and its regulator, respectively [142]. Both *dysc* and *slo* mutants also exhibit synaptic defects at the larval neuromuscular junction, which are rescued by mutating *jet* [142]. These findings raise the possibility that the influence of *jet* on circadian rhythms may extend beyond the molecular clock machinery.

#### 3.2.5. BRWD3 (Bromodomain and WD Repeat Domain Containing 3)

BRWD3 functions as a substrate receptor for CUL4–RING (CRL4) E3 ubiquitin ligase complexes and was identified in an RNAi screen for E3 ligases that mediate light-induced CRY degradation [143]. Similar to JET, BRWD3 binds to CRY in a light-dependent manner [143]. However, unlike *jet* silencing, the knockdown of *brwd3* attenuates light-evoked CRY degradation by proteasomes in S2 cells [143]. Further experiments showed that BRWD3 co-precipitates with other components of the CRL4 E3 ligase (namely, DDB1, CUL4A, CUL4B, and ROC1), and the BRWD3–CRL4 complex catalyzes the ubiquitination of CRY in vitro [143]. This contrasts with JET, which binds to but does not promote the ubiquitylation of CRY [143]. The study suggests that light during the day induces the formation of a protein complex consisting of TIM, CRY, BRWD3, and JET, causing BRWD3 and JET to ubiquitylate their respective substrates, CRY and TIM, and induce their degradation.

#### 3.2.6. MORGUE

MORGUE is a combined E2/E3 protein that harbours an F-box as well as a variant E2 conjugase domain [144,145]. MORGUE has been shown to physically associate with SkpA, a subunit of the SCF complex, as well as K48-linked polyubiquitin chains [146]. The overexpression of *morgue* in *tim*- but not *Pdf*-positive cells protects flies from LL-induced behavioural arrhythmicity [147]. Under LL conditions, *morgue* overexpression drives robust PER rhythms only in a subset of DN1 neurons and not in other clock neurons including the LN_v_s, indicating that *morgue* function in DN1s is critical for maintaining rhythmicity under LL [147]. MORGUE has been suggested to inhibit the CRY input pathway in DN1s, thus preventing the constant degradation of TIM and the consequent loss of rhythms under LL [147]. This potential relationship between MORGUE and the CRY input pathway is inferred from the observation that light-induced phase shifts are severely blunted in *morgue*-overexpressing flies, as they are in *cry* hypomorphs [147,148]. However, a molecular interaction between MORGUE and CRY has not been demonstrated thus far.

#### 3.2.7. CULLIN-3 (CUL3)

CULLIN-3 is a RING finger type E3 ubiquitin ligase that forms SCF complexes distinct from those containing SLIMB or JET, which are CUL1-based [149]. The silencing of *cul-3* in *cry*- or *Pdf*-expressing clock neurons leads to weakly rhythmic or arrhythmic behaviour [150]. The *tim*-specific overexpression of dominant-negative CUL3 mutants that cannot undergo neddylation also elicits arrhythmicity under DD [150]. A more recent study showed that CUL3 is required for light-induced clock resetting in CRY-deficient flies [151]. At the molecular level, *cul-3* knockdown flies exhibit dampened PER and TIM oscillations in the s-LN_v_s due to a lower peak expression of these proteins in the night [150]. TIM appears to be the major substrate of CUL3, as the dampening of TIM oscillations upon *cul-3* silencing occurs at a much faster rate than that of PER [150]. In the absence of PER, CUL3 associates with the hypophosphorylated form of TIM [150]. This is different from TIM/SLIMB complexes, which are formed in the presence of PER and consist of phosphorylated TIM [134]. Neddylation-deficient CUL3 mutants exhibit an accumulation of hyperphosphorylated TIM in the cytoplasm, suggesting that CUL3 promotes the degradation of either phosphorylated TIM or its hypophosphorylated form, the latter of which undergoes further phosphorylation [150]. However, despite the accumulation of TIM in neddylation-deficient CUL3 mutants, a subsequent study did not find an expected decrease in the ubiquitylation levels of TIM in these animals [152]. Only upon the concurrent inhibition of CUL3 and SLIMB activity is there a reduction in TIM ubiquitylation, suggesting that these two E3 ligases are partly redundant or act cooperatively [152]. CUL3/TIM association at the day–night transition temporally coincides with the detection of K48-linked polyubiquitination of TIM, a signal for proteasomal degradation [152]. Interestingly, the K48-linked polyubiquitination of the signaling protein Ci (Cubitus interruptus) has previously been shown to be favoured by CUL3-based SCF complexes, whereas SLIMB-based SCF complexes preferentially catalyze K11 linkages [153]. It remains to be seen whether the same holds true for the ubiquitylation of TIM by CUL3 and SLIMB, and how potential differences in Ub linkage affect the fate of TIM. In addition to its effects on TIM, CUL3 influences the accumulation of PDF in the axonal terminals of s-LN_v_s [133].

#### 3.2.8. TANGO10

TANGO10 is an adaptor protein for CUL3 and contains both BTB (broad-complex, tramtrack, and bric à brac) and BACK (BTB and C-terminal kelch) domains, which are important for protein–protein interactions [133]. Global loss-of-function mutations in *tango10* disrupt behavioural rhythmicity under DD, whereas the ubiquitous or pan-neuronal overexpression of wild-type *tango10* rescues this phenotype [133]. Suppressing *tango10* overexpression specifically in PDF neurons blocks the rescue, indicating that this gene is required in PDF neurons to maintain behavioural rhythms [133]. As expected, the silencing of endogenous *tango10* in PDF neurons also leads to behavioural arrhythmicity [133]. In global loss-of-function *tango10* mutants, the peak expression and oscillatory amplitude of PER and TIM are markedly reduced in the s-LN_v_s but not in other groups of pacemaker neurons in the brain [133]. Intriguingly, PDF accumulates in the dorsal axonal terminals of the s-LN_v_s of *tango10* mutants to aberrantly high levels [133]. TANGO10 co-localizes with PDF in the dorsal terminals and fluctuates in expression in a circadian fashion [133]. In *tango10* loss-of-function mutants, PDF neurons are hyperexcitable, potentially as a result of reductions in I_A_ potassium currents [133]. However, it is not known whether these changes in neuronal excitability are caused by, or the result of, perturbed molecular oscillations in the absence of *tango10.*

#### 3.2.9. FBXL4

FBXL4 is an F-box protein of the Fbls class, and as such possesses tandem leucine-rich repeats that are important for protein–protein interactions [58]. *Drosophila fbxl4* is expressed in the l-LN_v_s in a rhythmic manner, peaking in the middle (mRNA) or the late (protein) night [154]. FBXL4 physically interacts with and promotes the ubiquitination of the GABA_A_ receptor, resistant to dieldrin (RDL) [154]. This ubiquitylation event targets RDL for degradation [154]. In the l-LN_v_s, RDL abundance is rhythmic, reaching its peak in the late day and its nadir in the late night, anti-phasic to FBXL4 oscillations [154]. The daily fluctuation in RDL expression modulates the GABA sensitivity, and thus excitability, of l-LN_v_s [154]. Although *fbxl4* is dispensable for circadian rhythms at the behavioural level, as an output of the circadian clock (i.e., the *fbxl4* gene promoter contains E-box elements and is directly regulated by CLOCK:CYCLE), it is critical for modulating the onset and duration of sleep through its effects on RDL in the l-LN_v_s [154].

## 4. The N-Degron Pathway and Its Role in Circadian Rhythms

### 4.1. Overview of the N-Degron Pathway

The N-degron pathway was discovered in 1986 by Varshavsky and colleagues, who noted that certain N-terminal residues, and their post-translational modification, can dramatically affect the stability of a protein [155]. This destabilizing N-terminal residue is the main determinant of an N-degron, the degradation signal of this pathway, which also encompasses an unstructured N-terminal tail and an internal lysine residue serving as the site of subsequent ubiquitylation [156,157]. N-degrons are generated either through endoproteolytic cleavage, which exposes a new residue at the N-terminus, or by the post-translational modification of the N-terminal residue [157]. E3 ubiquitin ligases that recognize and bind to N-degrons are known as N-recognins [157,158].

There are four classes of eukaryotic N-degrons that are based on the characteristics of the N-terminal residue: the Arg/N-degrons, Ac/N-degrons, Pro/N-degrons, and fMet/N-degrons [157]. In Arg/N-degrons, the N-terminal residue is commonly an arginine, although it can also be a lysine, histidine, leucine, phenylalanine, tyrosine, tryptophan, isoleucine, or methionine that is followed by a bulky hydrophobic residue [155]. Other N-terminal residues such as asparagine (N), glutamine (Q), aspartate (D), glutamate (E), and cysteine (C) can also lead to the creation of an N-terminal arginine through deamidation (N, Q) or oxidation (C) followed by arginylation (C, D, E, N, and Q) [159]. Arginine is the structurally preferred degron for the UBR box-containing N-recognins [160]. In Ac/N-degrons, the N-terminus is acetylated to create a destabilizing residue [161]. The most frequently acetylated residues are serine and alanine, and to a lesser extent methionine, threonine, valine, and cysteine [161]. Pro/N-degrons contain a proline in, or immediately adjacent to, the N-terminus, whereas fMet/N-degrons carry a formylated methionine residue in the N-terminus [162,163]. The plethora of ways through which a destabilizing N-terminal residue may be created suggests that the N-degron pathway may play an important role in the degradation of many proteins.

The first proteins to be identified as N-recognins were those belonging to the ubiquitin ligase E3 component N-recognin (UBR) family [164]. The seven proteins that constitute this family, UBR1 to UBR7, all contain the UBR box, a conserved substrate recognition domain, but only four of them—UBR1, UBR2, UBR4, and UBR5—function as N-recognins specifically for the Arg/N-degron pathway [165,166]. Similar to other E3 ligases, UBR1, UBR2, and UBR5 also contain RING finger and/or HECT domains, whereas these domains are absent in UBR4 [165]. Of the four mammalian N-recognins, UBR1 and UBR2 are the most similar in sequence (46% identical) and substrate specificity [165]. In contrast, UBR4 and UBR5 have distinct structures and substrate specificities when compared to each other and to UBR1 and UBR2 [165]. Instead of UBR proteins, different E3 ligases are utilized by the other N-degron pathways as their N-recognins. TEB4 and NOT4, the latter a component of the CCR4–NOT complex, have been identified as N-recognins for the Ac/N-degron pathway [167,168]. The N-recognins that have been discovered for the Pro/N-degron and fMet/N-degron pathways are GID and PSH1, respectively [162,163].

### 4.2. The Roles of N-Recognins in Circadian Rhythms

Very few studies have addressed the potential role of the N-degron pathways and their associated N-recognins in the regulation of circadian rhythms. Varshavsky and colleagues demonstrated that serotonin N-acetyltransferase (AANAT), generally considered as the rate-limiting enzyme in the conversion of serotonin to melatonin, is degraded by the N-degron pathway in rodents [169]. While the acetylated form of rat AANAT is targeted by the Ac/N-degron pathway, the non-acetylated form is recognized by the Arg/N-degron pathway [169]. In contrast, human AANAT, which diverges from the rat homolog in its N-terminal sequence, has a much longer half-life and is not a substrate of the N-degron pathway [169]. These results suggest that the N-degron pathway accounts for the stark differences in the regulation of rodent and human AANAT.

In terms of N-recognins, only UBR1 and UBR4 have been implicated in the regulation of circadian rhythms [170,171,172]. In the case of UBR1, it was shown to induce the degradation of CONIDIAL SEPARATION 1 (CSP1), a *Neurospora* protein whose presence in the morning represses the transcription of evening-specific genes [172]. The phosphorylation of CSP1 triggers the recruitment and binding of UBR1, which, along with the E2 conjugase RAD6, promotes the rapid degradation of CSP1 [172]. The study suggests that this rapid turnover of CSP1 is essential for ensuring the tight coupling between first- and second-tier oscillations, driven by WHITE COLLAR COMPLEX (WCC), the core transcription factor of the *Neurospora* clock, and CSP1, a WCC-controlled gene, respectively [172].

More recently, UBR4 and its *Drosophila* homolog, POE (purity of essence), have been shown to affect circadian rhythms. UBR4 and POE are broadly expressed in the murine and fly brains, including virtually all SCN neurons and the PDF-positive LN_v_s [170]. A prior study demonstrated that the abundance of UBR4 in the SCN is both rhythmic and light-inducible, suggesting that it may regulate clock-timing processes or photic entrainment [171]. However, ablating *Ubr4* in all GABAergic neurons, and thus all SCN neurons, has no effect on free-running rhythms under DD or light-induced phase delays in mice [170]. Rather, *Ubr4* conditional knockout (cKO) mice are more susceptible to LL-induced arrhythmicity, exhibit shorter, dampened rhythms under LL, and entrain more rapidly and efficiently to shifted LD cycles mimicking acute and chronic jetlag conditions [170]. In comparison, silencing *poe* in PDF neurons results in a phenotype resembling the loss of *Pdf*: *poe* knockdown (KD) flies exhibit unstable rhythms as well as arrhythmic behaviour under DD [170,173]. In both the mouse and fly models, abolishing *Ubr4/poe* expression in central clock neurons suppresses levels of mPER2 or dPER [170]. The exact relationship between UBR4/POE and PER remains unclear, but the observations suggest that UBR4/POE is not acting as an N-recognin for mPER2/dPER.

Unexpectedly, the trafficking of circadian neuropeptides is perturbed in both animal models. *Ubr4* cKO mice exhibit a retention of AVP and VIP in the soma of SCN neurons, in contrast with wild-type controls, where these neuropeptides are primarily located in axonal projections [170]. In *poe* KD flies, PDF is similarly localized to the soma of s-LN_v_s and is less abundant in axonal projections [170]. Furthermore, the absence of UBR4 in HEK293T cells prevents ectopically expressed neuropeptide Y (NPY) from exiting the Golgi apparatus efficiently, indicating that the vesicular trafficking of cargo proteins is impaired at the level of Golgi export [170]. Importantly, UBR4 does not require its N-recognin activity to regulate vesicular trafficking, given that the phenotype of *UBR4* KO HEK293T cells is rescued by the overexpression of *UBR4* mutants in which the function of the UBR-box domain has been abolished [170]. Rather, UBR4 positively regulates the abundance of Coronin-7 (CRN7), which has been shown to mediate cargo export from the Golgi [170,174]. Boosting CRN7 expression in the absence of UBR4 rescues the trafficking deficit in cultured cells and the chronic jetlag phenotype in *Ubr4* cKO mice [170]. How UBR4 controls CRN7 abundance remains unclear, although there are data suggesting that it may be through a translational mechanism [170].

## 5. Concluding Remarks and Perspective: Ubiquitin Ligases—Going beyond Proteolysis

Great efforts have been directed towards finding ubiquitin ligases that target components of the core clock machinery for degradation, as their activity should also impact the key properties of circadian rhythms. To date, more than twenty E3 ligases are known to play a role in the regulation of circadian rhythms in mice and flies, mostly through the control of clock protein stability. A small handful of studies has illustrated potential non-proteolyic functions of E3 ligases within the circadian clock system. For example, the monoubiquitination of histones by DDB1–CUL4A negatively regulates E-box-dependent transcription through the recruitment of PER complexes [81]. TANGO10 regulates the density of I_A_ potassium currents in PDF neurons, potentially as a consequence of altered trafficking, stability, or function of potassium channels [133]. UBR4/POE regulates the trafficking of circadian neuropeptides through a novel mechanism that is independent of its canonical function as an N-recognin for the Arg/N-degron pathway [170]. These studies expand our views of the potential roles of E3 ligases in circadian timekeeping beyond their well-recognized involvement in the ubiquitin–proteasome system. The type of ubiquitination (mono-, multi-, or poly-) and the topology of the ubiquitin chain ultimately determine the fate of the ubiquitylated protein, be it degradation by proteasomes or lysosomes, trafficking to a new subcellular location, altered activity or protein binding, or the induction of cell signaling. Understanding the types of Ub linkages that a particular E3 ligase can catalyze will offer valuable insights into its potential role in circadian clock mechanisms. As suggested by the UBR4/POE study, some E3 ligases may have “moonlighting” functions that do not depend on their ability to ubiquitylate target proteins. For instance, they may serve as a scaffold for the assembly of protein complexes. New insights may be uncovered by exploring the potential non-canonical functions of E3 ligases. Lastly, even though the timely turnover of core clock proteins undoubtedly plays a crucial role in generating proper circadian rhythms, other aspects of cellular physiology also contribute to a temporally precise clock. The ubiquitination and proteolysis of factors that influence neuronal excitability, inter- and intra-cellular communication, protein trafficking, transcription, translation, and cellular metabolism also merit the attention of researchers as they advance our understanding of the role of E3 ligases in circadian rhythms.

## Data Availability

Not applicable.
